# *Salmonella* Infection Induces Orchitis and Disrupts the Blood–Testis Barrier, Leading to Spermatogenic Disorders in Mice

**DOI:** 10.3390/microorganisms14081862

**Published:** 2026-08-21

**Authors:** Yingchao Li, Qian Ma, Chenyang Shi, Qirui Zang, Yaolong Song, Mingshuai Chen, Binhuan Ma, Panpan Tong, Zhanqiang Su, Yi Zhang, Shicheng Wan, Aili Aierken, Mengfei Zhang

**Affiliations:** 1 College of Veterinary Medicine, Xinjiang Agricultural University, Urumqi 830052, China; liyingchao0409@163.com (Y.L.); 15339306406@163.com (Q.M.); 17634384908@163.com (C.S.); 19199130252@163.com (Q.Z.); 17726785228@163.com (Y.S.); chenms416@outlook.com (M.C.); 15750679553@163.com (B.M.); tongpanpan@xjau.edu.cn (P.T.); szq00009@163.com (Z.S.); zydy052@163.com (Y.Z.); 2 Urumqi Field Scientific Observation and Research Station for Animal Diseases, Ministry of Agriculture and Rural Affairs, Urumqi 830052, China; 3 Xinjiang Key Laboratory of New Drug Research and Development for Herbivorous Animals, Urumqi 830052, China; 4 Xinjiang Regional Key Laboratory of Clinical Veterinary Medicine Research, Urumqi 830052, China; 5 Herbivorous Animal Biomedicine and Diagnostic Technology Engineering Research Center, Xinjiang Uygur Autonomous Region, Urumqi 830052, China; 6 College of Veterinary Medicine, Northwest A&F University, Yangling 712100, China; shicheng_wan@nwafu.edu.cn; 7 State Key Laboratory of Pathogenesis, Prevention and Treatment of High Incidence Diseases in Central Asia, Xinjiang Medical University, Urumqi 830011, China; alijan@xjmu.edu.cn

**Keywords:** *Salmonella*, orchitis, BTB, spermatogenic dysfunction

## Abstract

This study investigated the pathological processes by which two *Salmonella* strains induce orchitis and impair spermatogenesis in mice, with emphasis on inflammation and blood–testis barrier (BTB) integrity. Thirty male Kunming mice were randomly assigned to the human-derived *Salmonella enterica* serovar Enteritidis H71 group, the sheep-derived *Salmonella enterica* serovar Agona W42 group, or the phosphate-buffered saline control group (n = 10 per group). An acute orchitis model was established by intrascrotal injection. Histopathological examination revealed marked testicular and epididymal lesions, disruption of the spermatogenic epithelium, and reduced sperm abundance in infected mice. Transcriptomic analysis identified 4546 differentially expressed genes shared by the two infected groups and showed enrichment of the Toll-like receptor (TLR), nuclear factor kappa B (NF-κB), and mitogen-activated protein kinase (MAPK) signaling pathways. Real-time quantitative PCR further showed increased expression of interleukin 6 (*Il6*), interleukin 1 beta (*Il1b*), and tumor necrosis factor (*Tnf*), accompanied by reduced expression of tight junction protein 1 (*Tjp1*), occludin (*Ocln*), and synaptonemal complex protein 3 (*Sycp3*) in infected mice (*p* < 0.05), except for *Tjp1* in the W42 group. These findings indicate that *Salmonella*-induced inflammatory activation is associated with BTB disruption and impaired spermatogenesis, providing a basis for further investigation of bacterial orchitis and zoonotic reproductive risks.

## 1. Introduction

Male reproductive disorders are a major factor limiting the efficiency and profitability of animal husbandry [1]. Among these disorders, orchitis caused by bacterial infections has been observed to substantially reduce or completely inhibit spermatogenic function [2]. *Salmonella* is an important zoonotic pathogen [3] that causes clinical or subclinical infections (salmonellosis) in humans and different economically important animals, including cattle, sheep, pigs, and poultry [4]. After a bacterial infection, the pathogen can spread through the bloodstream to organs such as the testes, colonize them, and cause orchitis, potentially leading to irreversible reproductive damage and economic losses in livestock farming [5,6]. Although there is extensive research on *Salmonella* in intestinal and systemic infections, studies on the specific molecular pathogenesis that damages testicular tissue and disrupts the spermatogenic microenvironment in the veterinary field are lacking.

BTB is a key structure that maintains immune function and the stability of the spermatogenic microenvironment. Furthermore, the functional integrity of BTB is crucial for normal spermatogenesis [7]. Previous studies have indicated that pathogenic infection and inflammation-induced stress can stimulate innate immune signaling pathways in the testes, including the Toll-like receptor/nuclear factor kappa B pathway, which induces the release of pro-inflammatory cytokines, disrupting the tight junctions between Sertoli cells [8,9]. The downregulation of tight junction core proteins (ZO-1 and Occludin) is a hallmark of BTB dysfunction and has been associated with the degeneration of the spermatogenic epithelium [10]. Current veterinary research on bacterial orchitis primarily focuses on specific pathogens such as *Brucella*, while molecular mechanisms underlying *Salmonella* infection, a common pathogen with a wide host range, as well as its induction of orchitis, have not been comprehensively studied. Moreover, the virulence and tissue tropism in hosts may vary with *Salmonella* strains, and investigating how these differences manifest in the testes is crucial for comprehending bacteria’s pathogenic characteristics and for prevention and control.

This study analyzed two *Salmonella* strains from different hosts [sheep-derived *Salmonella enterica* serovar Agona (W42) and human-derived *Salmonella enterica* serovar Enteritidis (H71)], which were administered to mice via intrascrotal injection to establish an acute orchitis model to investigate their underlying pathogenic mechanisms. Using this infection model and transcriptome sequencing, a systematic comparative analysis of the infected groups (W42 and H71) and the wild-type control group (WT) was carried out. Furthermore, this study identified the similarities and differences in the innate immune and inflammation-related signaling pathways activated in the testes after infection, the modulatory effects of inflammation on key BTB tight junction protein gene levels, and the effect of infection on key spermatogenesis genes and pathways. The findings will identify the core molecular pathways by which *Salmonella* infection causes orchitis and spermatogenic disorders; the pathogenic characteristics of strains from different sources will be compared; and a reference will be provided to prevent and control *Salmonella*-induced orchitis in livestock, improving reproductive health in animal husbandry.

## 2. Materials and Methods

### 2.1. Experimental Strain

The H71 strain was isolated in September 2024 from a fecal sample of a 1-year-old boy at the Urumqi Center for Disease Control and Prevention in Xinjiang, China. The W42 strain was isolated from a rectal swab at a sheep farm in Urumqi, Xinjiang, China, in December 2019. 16S rRNA gene sequencing preliminarily identified both isolates as *Salmonella* spp. Their serovars were subsequently determined by whole-genome sequencing and SalmonellaTypeFinder as *Salmonella enterica* serovar Enteritidis for H71 and *Salmonella enterica* serovar Agona for W42.

### 2.2. Whole-Genome Sequencing of Salmonella

Total DNA was extracted from the sample for PCR amplification of the bacterial 16S rRNA gene using specific tag primers 27F (5′-AGAGTTTGATCCTGGCTCAG-3′) and 1492R (5′-GGTTACCTTGTTACGACTT-3′). The PCR products were purified and subjected to 16S rRNA sequencing for preliminary bacterial identification. Subsequently, whole-genome sequencing was performed on the Illumina platform. The assembled genome sequences were analyzed using SalmonellaTypeFinder (VFAnalyzer v5) for serotype prediction, SRST2 for MLST analysis, PlasmidFinder for plasmid identification, and the Comprehensive Antibiotic Resistance Database (CARD) for antimicrobial resistance gene prediction. To generate high-quality representative sequences, the raw data were subjected to quality control, sequence assembly, and denoising based on the platform’s standard workflow. The obtained sequences were further annotated for species taxonomy by database comparison. SalmonellaTypeFinder was employed to predict the serotype and combined with SRST2 to determine MLST typing. The assembled genes were uploaded to PlasmidFinder to evaluate the plasmid content of the strains. The antibiotic resistance gene database (https://card.mcmaster.ca/) was employed to predict antibiotic-resistance genes in the genome [11,12].

### 2.3. Experimental Strains and Animal Infection

Kunming mice (n = 30, male, age = 6–7 weeks) were randomly divided into the following 3 groups (n = 10 mice/group): two infection (H71 and W42) groups and a WT (PBS control) group. All animal protocols were approved by the Ethics Committee of Xinjiang Agricultural University (No. 20240519) and followed the guidelines of ‘Animal Research: In Vivo Experiments Reporting’. Both H71 and W42 strains were cultured in Rappaport–Vassiliadis (RV) broth and centrifuged at 12,000 rpm for 10 min using a refrigerated centrifuge. The bacterial pellet was resuspended in phosphate-buffered saline (PBS) to adjust the bacterial concentration to 1 × 10^8^ CFU/mL. Mice in the infection groups received 0.1 mL of bacterial suspension via intrascrotal injection. Mice in the WT group received an equal volume of sterile PBS via the same intrascrotal injection procedure, serving as the mechanical stimulation control. Testes and epididymides were collected 24 h after injection, fixed in 4% paraformaldehyde, and stained with hematoxylin and eosin (H&E). Testicular parenchyma was immersed in Total RNA extraction reagent (TaKaRa, Hangzhou, China), homogenized, and stored at −80 °C for subsequent transcriptome sequencing.

### 2.4. Transcriptome Sequencing

Total RNA was isolated from testicular tissues collected from three independent mice per group. Each mouse was treated as one biological replicate. RNA-seq libraries were constructed separately for each biological replicate and sequenced on the Illumina platform at Beijing Novogene Co., Ltd. (Beijing, China). Subsequently, data quality control and bioinformatics analyses were performed. Lastly, GO functional and KEGG pathway enrichment analyses of DEGs were conducted [11,12].

### 2.5. Quantitative Real-Time PCR

Total RNA was isolated from mouse testes with Total RNA extraction reagent on ice, mixed with a 20 μL reverse transcription reaction system, and reverse-transcribed into cDNA on a PCR instrument. Based on the KEGG pathway enrichment results and their biological relevance to inflammation, blood–testis barrier integrity, and spermatogenesis, *Il6*, *Il1b*, *Tnf*, *Tjp1*, *Ocln*, and *Sycp3* were selected for RT-qPCR validation. Real‑time qPCR was performed using the BioEasy SYBR Kit (Hangzhou Bioer Technology Co., Ltd., Hangzhou, China). The relative expression levels of these genes were subsequently calculated. All samples were tested in at least three biological replicates [13,14].

### 2.6. Statistical Analysis

All experiments were performed with at least three independent biological replicates. Data are presented as the mean ± standard deviation (SD). Statistical analyses were performed using GraphPad Prism 8.0.2. Differences among multiple groups were analyzed using one-way analysis of variance (ANOVA) followed by Tukey’s multiple comparisons test. A value of *p* < 0.05 was considered statistically significant.

## 3. Results

### 3.1. Second-Generation Sequencing Results of Salmonella

The SalmonellaTypeFinder was used to identify the serotype of H71, which was *Salmonella enterica* serovar Enteritidis, with the antigen formula O: 9, H1: g, m, and H2: -. Furthermore, its resistance genes included aph(6)-Id, aph(3″)-Ib, tet(A), sul2, and blaTEM-1. The W42 serotype was *Salmonella enterica* serovar Agona, with the antigen formula O:4, H1 antigens: f,g,s, H2 antigen: -. Its predicted resistance genes were qnrS1, tet(A), sul3, blaTEM-1, aph(3″)-Ib, aph(6)-Id, dfrA14, and fosA7.2.

### 3.2. Salmonella Infection of Mouse Testes and Epididymis

**Figure 1:**
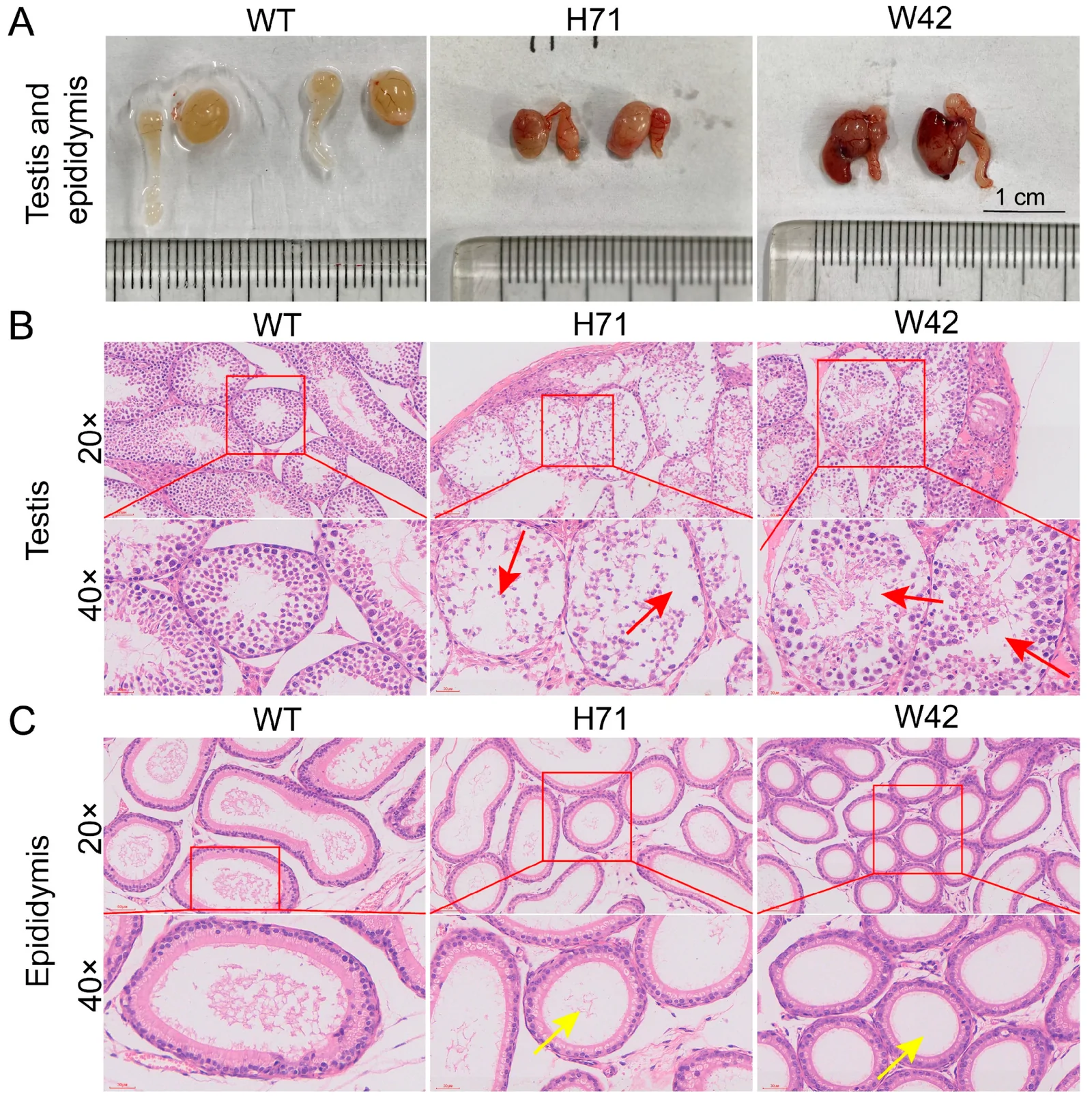
Histopathological observation of *Salmonella*-induced orchitis in mice. (**A**) Gross appearance of testes and epididymides from the WT (PBS-injected control), H71, and W42 groups. (**B**) Histopathological sections of testes and (**C**) epididymides from the WT (PBS-injected control), H71, and W42 groups (20× and 40×).

Mice received intrascrotal injections of 0.1 mL of H71 or W42 bacterial suspension. Gross examination showed marked hemorrhagic changes in the testes and epididymides of both infected groups, whereas no comparable gross alterations were observed in the WT group (Figure 1A). The hemorrhagic appearance was slightly more evident in the W42 group than in the H71 group. Histological examination showed that the WT group retained a relatively regular testicular structure. In contrast, both infected groups exhibited disruption of the seminiferous tubule architecture, disorganization and loss of spermatogenic cells, and visible alterations in the spermatogenic epithelium, as indicated by the red arrows in Figure 1B. Examination of the epididymides further showed reduced sperm abundance in the luminal regions of the H71 and W42 groups compared with the WT group, as indicated by the yellow arrows in Figure 1C. Collectively, these findings showed that both strains induced acute pathological changes in the testes and epididymides and were associated with impaired spermatogenic function.

### 3.3. Gene Expression Level Distribution of Salmonella-Induced Orchitis in Mice

**Figure 2:**
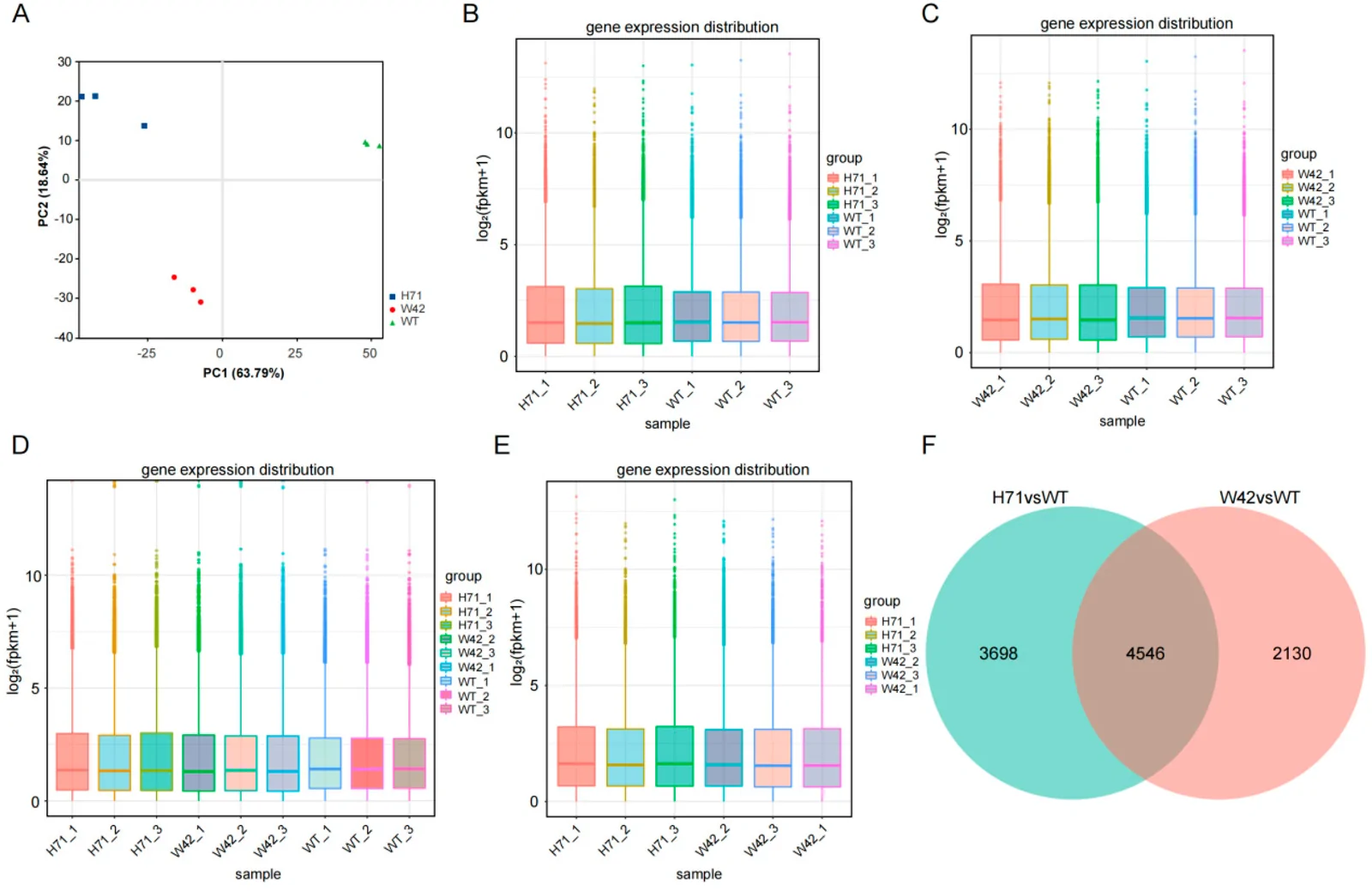
Gene expression level distribution of *Salmonella*-induced orchitis in mice. (**A**) PCA plot. (**B**) Gene expression distribution between H71 vs. WT groups, (**C**) W42 vs. WT groups, (**D**) H71, W42, and WT groups, and (**E**) H71 vs. W42 groups. (**F**) Venn diagram of H71 vs. WT and W42 vs. WT.

Transcriptome sequencing was performed using testicular tissues from the WT, H71, and W42 groups. Principal component analysis was conducted to evaluate the overall relationships among the transcriptomic profiles of the samples. The PCA plot showed separation among the WT, H71, and W42 groups, indicating that the overall gene expression profiles differed among the three experimental conditions (Figure 2A). The H71 and W42 samples also occupied different positions in the PCA plot, showing that the transcriptional changes associated with the two strains were not completely identical. Boxplots were generated to display the distributions of gene expression levels across the individual samples and comparison groups (Figure 2B–E). These plots provided an overview of the expression profiles used for subsequent differential expression analysis. Venn analysis of the H71 vs. WT and W42 vs. WT comparisons identified 4546 shared DEGs (Figure 2F). In addition, 3698 DEGs were detected only in the H71 vs. WT comparison, whereas 2130 DEGs were detected only in the W42 vs. WT comparison. Thus, the transcriptional responses to H71 and W42 included both a substantial common set of changes and strain-associated differences.

### 3.4. Differential Gene Expression Analysis of Salmonella-Induced Orchitis in Mice

**Figure 3:**
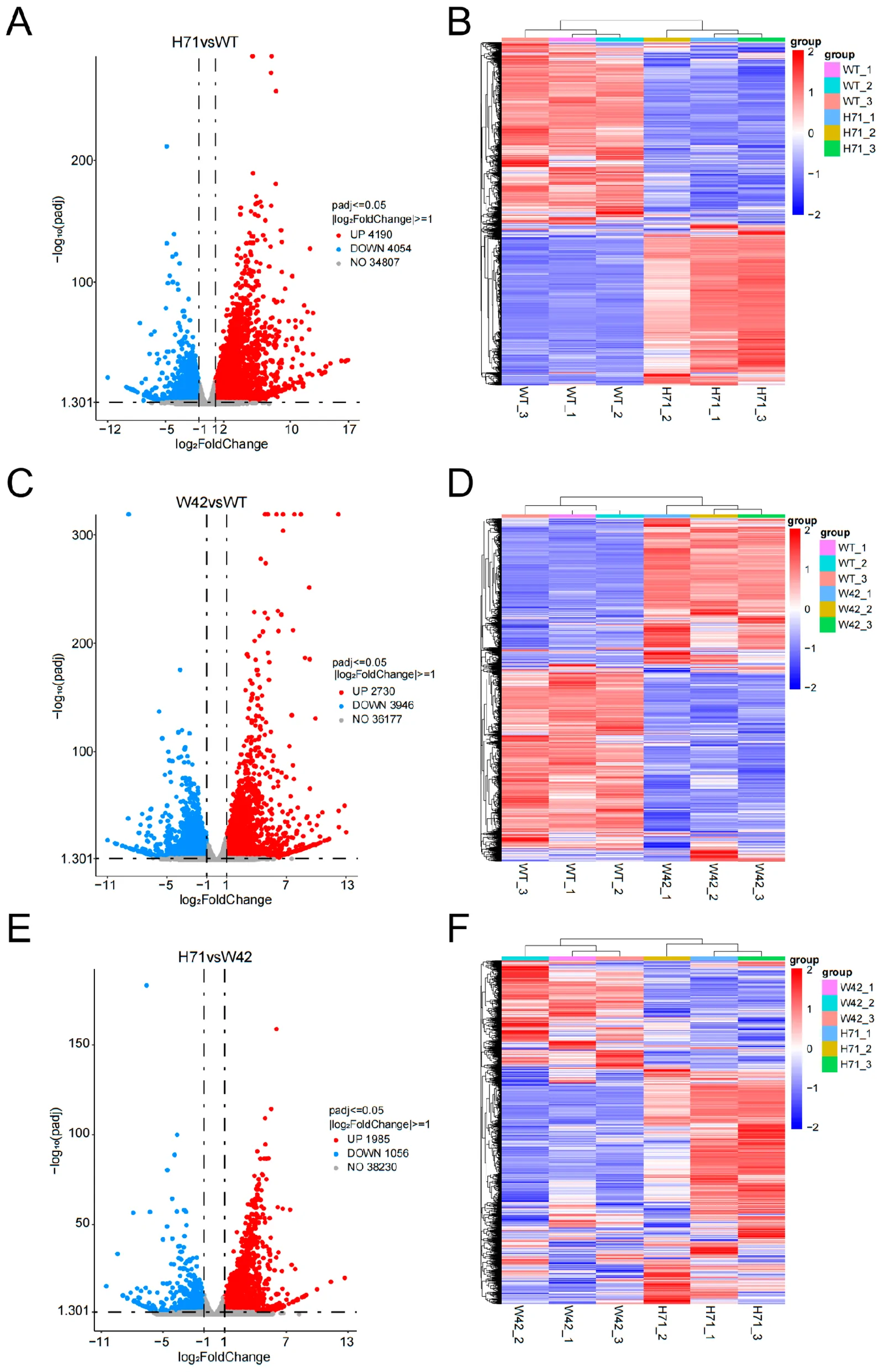
Differential gene expression analysis of *Salmonella*-induced orchitis in mice. (**A**) Volcano plot of H71 vs. WT. (**B**) Clustered heatmap of H71 vs. WT. (**C**) Volcano plot of W42 vs. WT. (**D**) Clustered heatmap of W42 vs. WT. (**E**) Volcano plot of H71 vs. W42. (**F**) Clustered heatmap of H71 vs. W42.

Differential expression analysis was performed using thresholds of |log_2_FoldChange| > 1 and adjusted *p* < 0.05. In the H71 vs. WT comparison, 8244 DEGs were identified, including 4190 upregulated and 4054 downregulated genes, whereas 34,807 genes did not meet the significance threshold (Figure 3A). The corresponding clustered heatmap showed distinct expression patterns between the H71 and WT samples (Figure 3B). In the W42 vs. WT comparison, a total of 6676 DEGs were detected, including 2730 upregulated and 3946 downregulated genes, while 36,177 genes were classified as non-significant (Figure 3C). The corresponding heatmap also showed different gene expression patterns between the W42 and WT samples (Figure 3D). Direct comparison between H71 and W42 identified 3041 DEGs, comprising 1985 genes with higher expression and 1056 genes with lower expression in H71 relative to W42; 38,230 genes were non-significant (Figure 3E). The corresponding clustered heatmap further displayed differences in gene expression patterns between the two infected groups (Figure 3F). These results showed that both strains produced extensive transcriptional changes relative to the WT group and that substantial differences were also present between H71 and W42.

### 3.5. GO Functional Enrichment Analysis of Salmonella-Induced Orchitis in Mice

**Figure 4:**
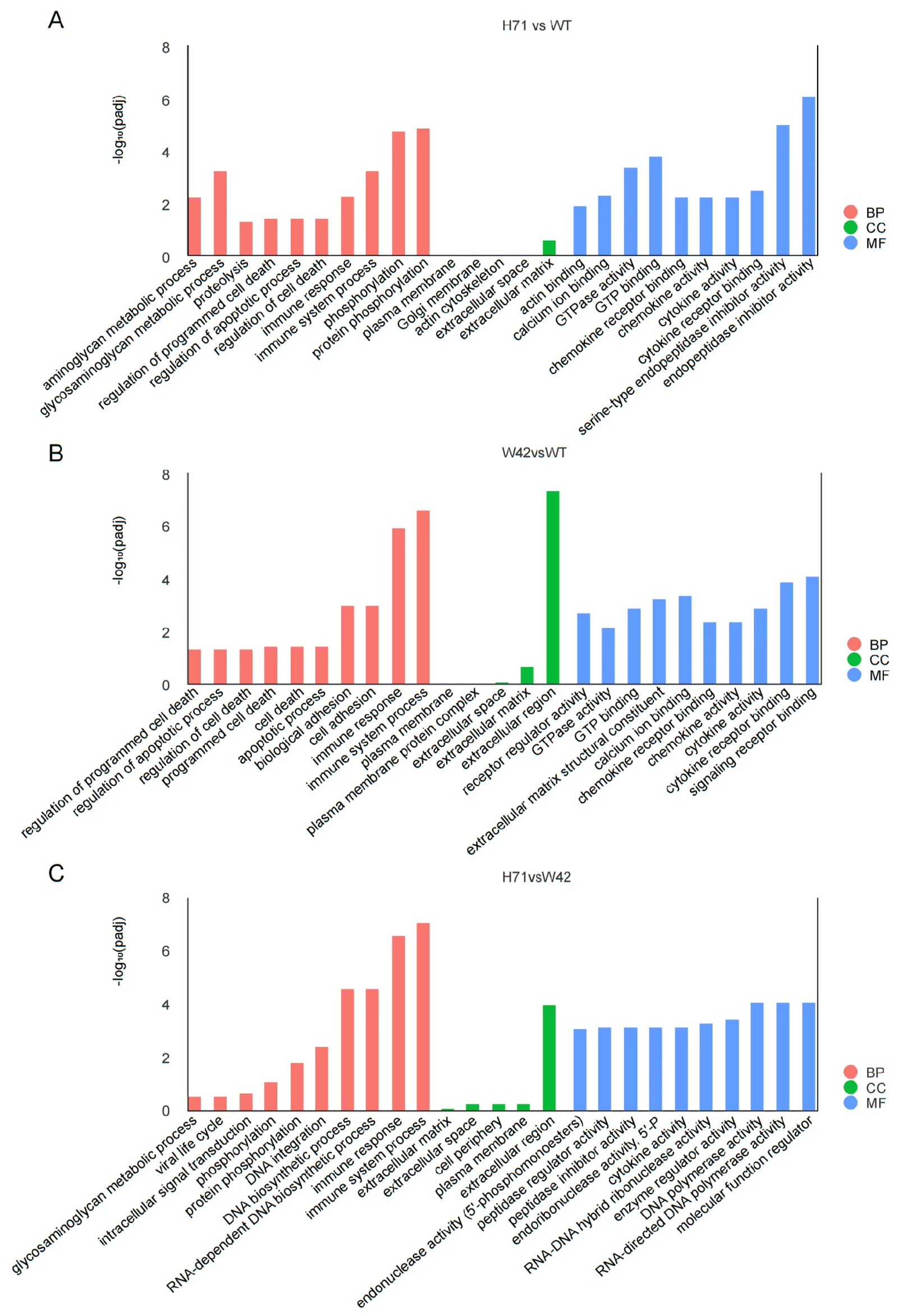
GO functional enrichment analysis of *Salmonella*-induced orchitis in mice. (**A**) GO functional enrichment analysis of H71 vs. WT, (**B**) W42 vs. WT, and (**C**) H71 vs. W42.

GO functional enrichment analysis was performed to characterize the biological functions associated with the identified DEGs. In both the H71 vs. WT and W42 vs. WT comparisons, the enriched biological-process categories mainly included immune system processes and immune responses, indicating that immune-related functions were prominently represented among the DEGs in both infected groups. These common enrichment patterns showed that the two *Salmonella* strains were associated with several overlapping biological functions in mouse testicular tissue. In the H71 vs. WT comparison, additional enriched terms included apoptosis-related regulation, glycosaminoglycan metabolic processes, phosphorylation-related processes, and other cellular regulatory functions (Figure 4A). In the W42 vs. WT comparison, the enriched categories included immune-related processes, apoptotic processes, regulation of cell death, cytokine-associated functions, and extracellular or membrane-associated components (Figure 4B). These results showed that immune responses, apoptosis-related processes, cytokine-associated functions, phosphorylation-related processes, and extracellular or membrane-associated functions were among the major categories associated with the identified DEGs. Although the H71 vs. WT and W42 vs. WT comparisons shared several enriched categories, the specific enrichment profiles were not completely identical. The H71 vs. W42 comparison further identified differences in biological-process, cellular-component, and molecular-function categories (Figure 4C). Taken together, the GO enrichment results showed that H71 and W42 shared several infection-associated functional categories while also exhibiting partially distinct enrichment patterns in the testicular transcriptome.

### 3.6. KEGG Pathway Enrichment Analysis of Salmonella-Induced Orchitis in Mice

**Figure 5:**
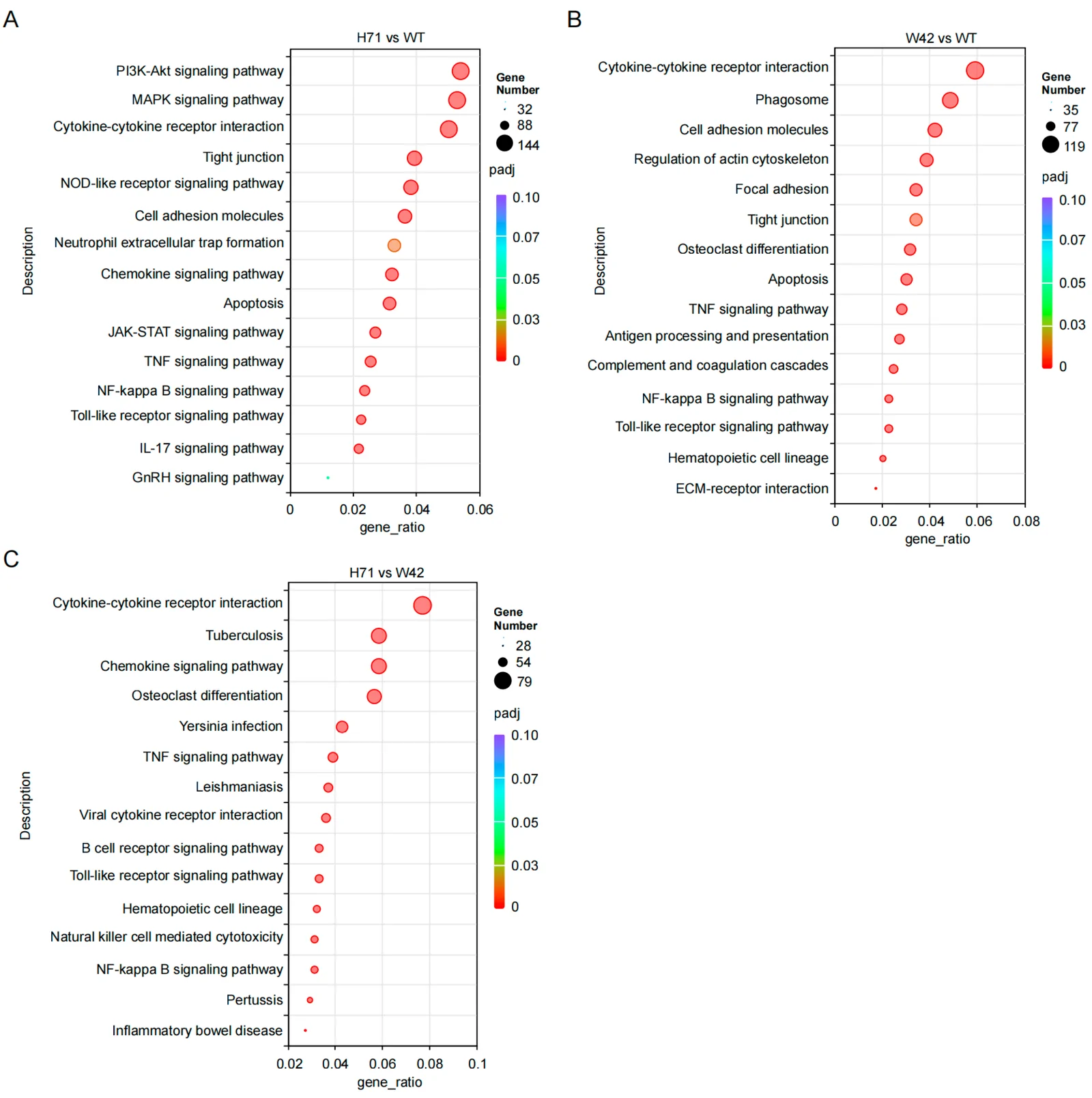
KEGG pathway enrichment analysis of *Salmonella*-induced orchitis in mice. (**A**) KEGG pathway enrichment analysis of H71 vs. WT, (**B**) W42 vs. WT, and (**C**) H71 vs. W42.

KEGG pathway enrichment analysis identified multiple pathways related to inflammatory signaling, innate immunity, apoptosis, cell adhesion, and tight junction-associated functions. In the H71 vs. WT comparison, DEGs were enriched in the PI3K–Akt, MAPK, cytokine–cytokine receptor interaction, NOD-like receptor, chemokine, JAK–STAT, TNF, NF-κB, and Toll-like receptor signaling pathways (Figure 5A). Enrichment was also observed in the tight junction, cell adhesion molecule, apoptosis, neutrophil extracellular trap formation, and IL-17 signaling pathways. In the W42 vs. WT comparison, enriched pathways included cytokine–cytokine receptor interaction, phagosome, regulation of the actin cytoskeleton, focal adhesion, tight junction, apoptosis, TNF signaling, antigen processing and presentation, complement and coagulation cascades, NF-κB signaling, and Toll-like receptor signaling (Figure 5B). Cell adhesion molecules, hematopoietic cell lineage, and extracellular matrix–receptor interaction were also represented among the enriched pathways. Several pathways were identified in both infection-versus-control comparisons, including Toll-like receptor signaling, NF-κB signaling, TNF signaling, apoptosis, and tight junction-associated pathways. This overlap was consistent with the substantial number of shared DEGs identified by the Venn analysis. However, the overall sets of enriched pathways were not identical between H71 and W42. Direct comparison between H71 and W42 showed enrichment in cytokine–cytokine receptor interaction, chemokine signaling, TNF signaling, Toll-like receptor signaling, NF-κB signaling, B-cell receptor signaling, natural killer cell-mediated cytotoxicity, and hematopoietic cell lineage, among other pathways shown in Figure 5C. These findings indicated that the two strains shared multiple immune- and inflammation-related responses while also differing in several pathway-level changes.

### 3.7. RT-qPCR Validation

**Figure 6:**
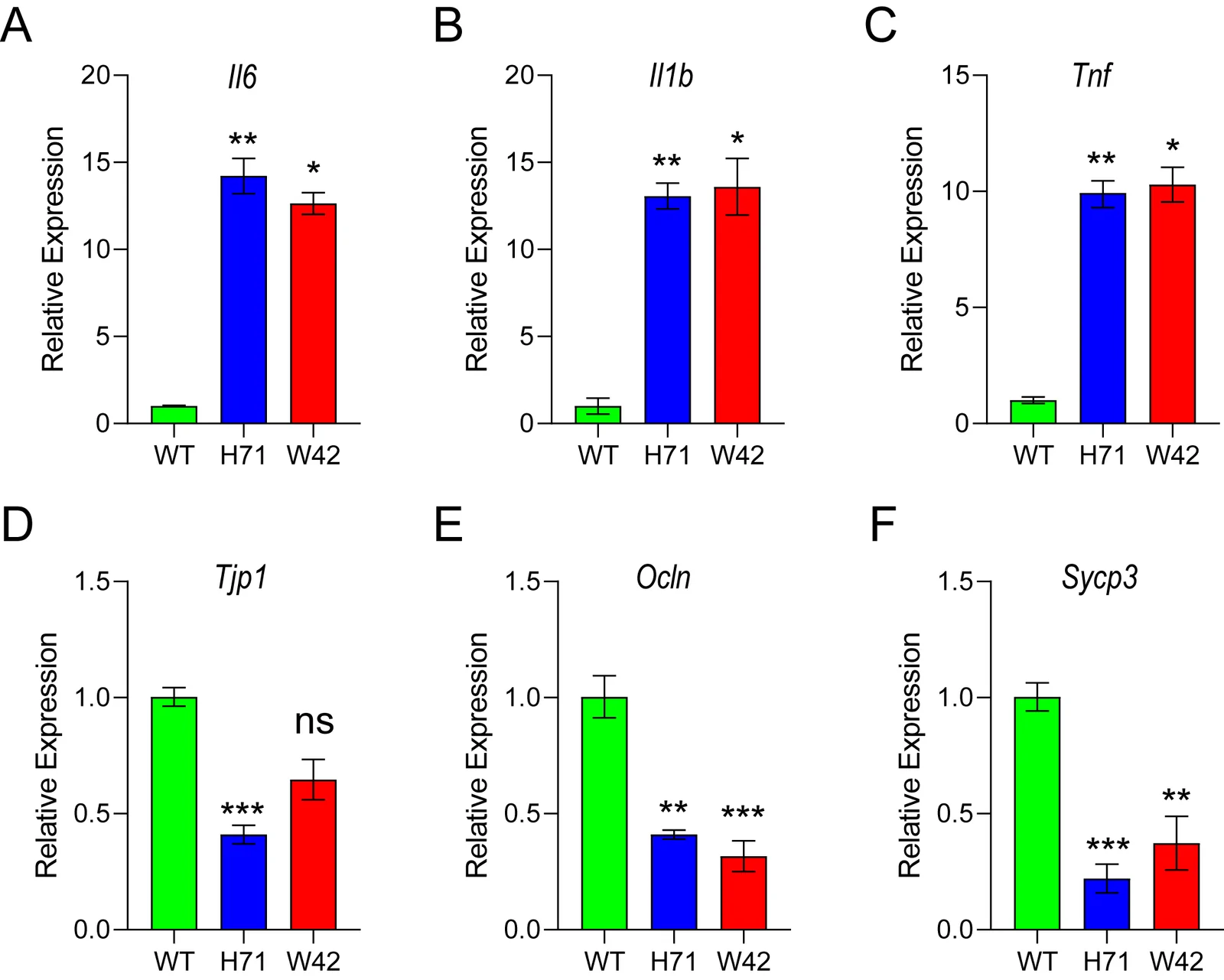
RT-qPCR validation of *Salmonella*-induced orchitis in mice. (**A**) Relative expression of *Il6*, (**B**) *Il1b*, (**C**) *Tnf*, (**D**) *Tjp1*, (**E**) *Ocln*, and (**F**) *Sycp3*. * *p* < 0.05; ** *p* < 0.01; *** *p* < 0.001; ns represents no significant difference.

Representative genes associated with inflammation-related pathways, BTB integrity, and spermatogenesis were selected for RT-qPCR validation. Compared with the WT group, *Il6* mRNA expression was significantly increased in both the H71 and W42 groups (Figure 6A). Similarly, *Il1b* and *Tnf* expression levels were significantly elevated in both infected groups (Figure 6B,C). These results confirmed increased expression of the three examined inflammatory cytokine genes following infection with either strain. The expression patterns of tight junction-related genes were also examined. *Tjp1* mRNA expression was significantly reduced in the H71 group compared with the WT group, whereas the reduction observed in the W42 group did not reach statistical significance (Figure 6D). *Ocln* expression was significantly decreased in both infected groups (Figure 6E). In addition, the meiosis-associated gene *Sycp3* was significantly downregulated in both the H71 and W42 groups relative to the WT group (Figure 6F). Overall, the RT-qPCR results showed increased expression of inflammatory cytokine genes together with reduced expression of genes associated with tight junction integrity and spermatogenesis. The directions of these changes were consistent with the transcriptomic and pathway enrichment results.

## 4. Discussion

**Figure 7:**
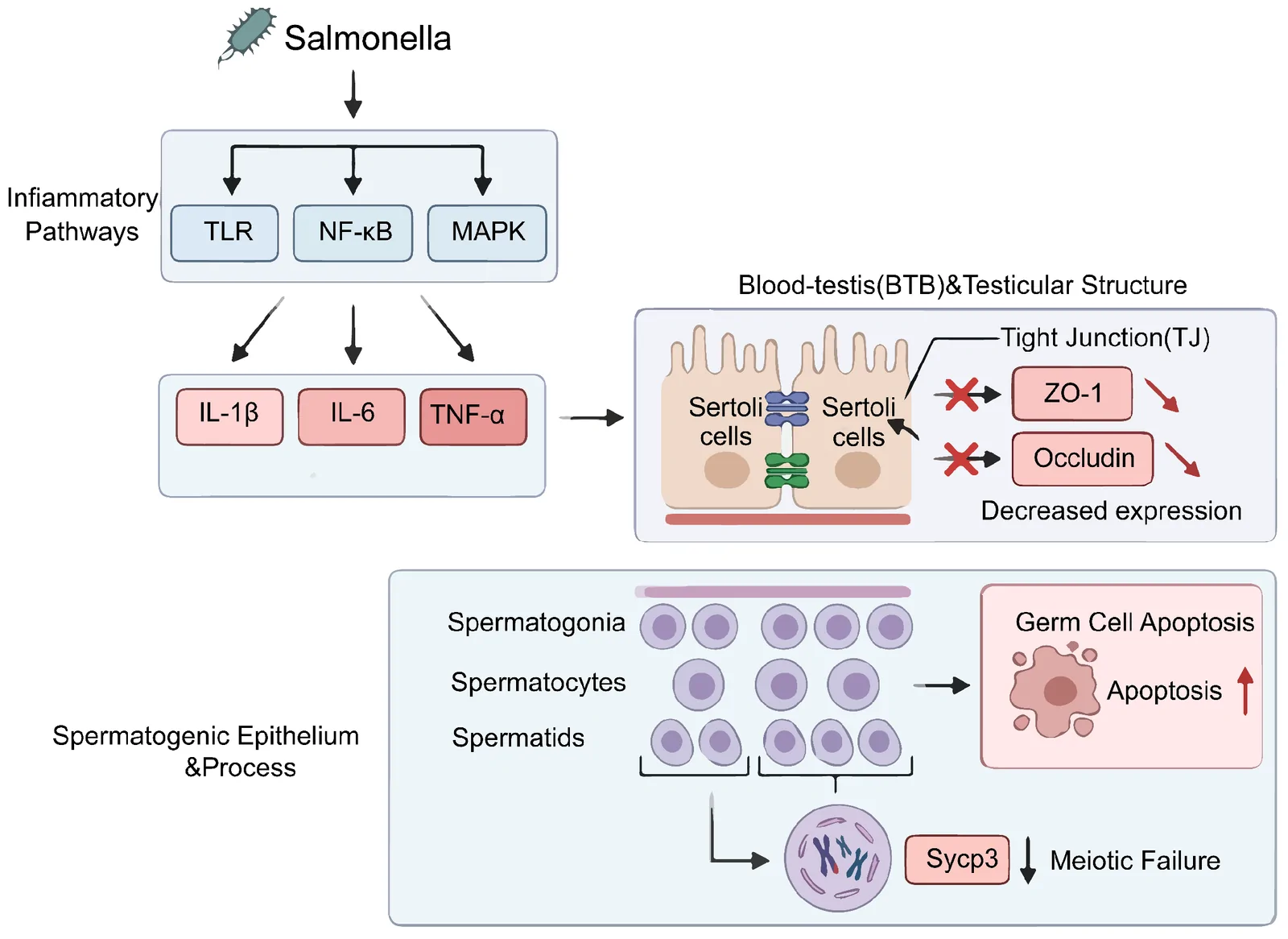
Schematic diagram of the proposed mechanism underlying *Salmonella*-induced orchitis, blood–testis barrier (BTB) disruption, and spermatogenic dysfunction in mice.

This study developed an acute orchitis mouse model by intrascrotal administration of *Salmonella* H71 and W42 strains to identify the key molecular mechanisms underlying the *Salmonella*-induced disruption of BTB, leading to spermatogenic disorders. The mechanism of *Salmonella*-induced orchitis in mice (Figure 7) demonstrated that BTB disruption impaired spermatogenesis. Histopathological examination, transcriptome sequencing, GO and KEGG enrichment analyses, and RT-qPCR validation consistently indicated that *Salmonella* infection was associated with inflammatory responses, alterations in BTB-related gene expression, and changes in spermatogenesis-associated genes. These complementary approaches provided consistent evidence supporting the overall biological changes observed in the infected testes and strengthened the reliability of the transcriptomic findings. The study demonstrated that H71 and W42 infections were associated with significant enrichment of classical inflammatory signaling pathways, and the transcriptomic results were further supported by RT-qPCR validation, which demonstrated increased expression of *Il6*, *Il1b*, and *Tnf* in both infected groups. The agreement between transcriptome sequencing and RT-qPCR indicates that inflammatory activation represented one of the major molecular responses induced following *Salmonella* infection in the testes. Similar inflammatory responses have also been reported in bacterial orchitis caused by other pathogens, suggesting that excessive local inflammation is a common feature of infectious testicular injury. Including, TLR, NF-κB, and MAPK signaling in testicular tissue (such as TLR/NF-κB/MAPK), which upregulates pro-inflammatory factors (*Il6* and *Tnf*), consistent with the testicular immunopathology observed after various pathogen infections [15,16]. The excessive inflammatory response compromises BTB integrity; in the present study, reduced expression of *Tjp1* and *Ocln* was consistent with the enrichment of tight junction-associated pathways identified by transcriptome sequencing. Together with the histopathological alterations observed in the seminiferous epithelium, these findings suggest that inflammatory responses and changes in tight junction-related genes occurred simultaneously during acute infection. However, direct assessment of BTB permeability or protein localization was beyond the scope of the present study and should be addressed in future investigations, as evidenced by substantial downregulation of tight junction protein (*Tjp1* and *Ocln*) transcription levels, consistent with mechanisms of epithelial barrier disruption during inflammatory conditions in organs such as the gut [17,18]. Loss of barrier function disrupts the immune-rich microenvironment of spermatogenic cells, promotes apoptosis, and induces spermatogenic cell shedding by altering the expression of cell adhesion molecules [19]. *Sycp3* encodes a major structural component of the synaptonemal complex and is essential for meiotic chromosome organization. Its reduced expression therefore suggests impaired meiosis and spermatogenic dysfunction. Here, it was found that *Salmonella* infection downregulated the meiotic core marker *Sycp3*, suggesting that spermatogenesis was substantially inhibited [20]. Although both *Salmonella* strains promoted similar pathological axes, transcriptomic data showed differences in certain downstream pathways they regulate, which might be associated with their different serotypes and potential virulence factor profiles [21], warranting further investigation. *Salmonella* infection impaired spermatogenesis via the ‘inflammatory activation–barrier disruption–spermatogenesis inhibition axis’. These results provide a molecular basis for understanding the pathogenesis of bacterial orchitis, including *Salmonella*-induced cases, and mainly emphasize early control of inflammation to protect male reproductive function. These results also provide novel theoretical targets for preventing and controlling *Salmonella*-related reproductive disorders in livestock and furnish experimental evidence for evaluating the potential cross-species risk of these zoonotic pathogens to the male reproductive system.

In addition to inflammatory pathways, infection significantly increases local oxidative stress in the testes, which may be related to excessive reactive oxygen species (ROS) production induced by the inflammatory cytokine storm [22,23]. In Sertoli and germ cells, the accumulation of ROS can further impair mitochondrial function, thus exacerbating apoptosis [24,25]. Furthermore, ROS accumulation directly affects tight junction proteins’ stability through oxidative modifications, which synergistically disrupt BTB and inflammatory responses [26]. Infection can also disrupt the paracrine signaling network between testicular Sertoli cells and germ cells, as well as alter the expression of key supportive factors, such as glial cell line-derived neurotrophic factor, which could also explain the inhibition of germ cell differentiation [27,28].

This study has several limitations. The acute orchitis model was established by intrascrotal injection, which enabled controlled and reproducible local infection but did not fully mimic the natural route of *Salmonella* infection. Further studies using natural infection routes, such as oral or systemic infection, as well as naturally infected hosts, are therefore needed. Because susceptibility and host responses to *Salmonella* may differ among animal species, the findings obtained in mice should be further validated in target livestock species, particularly sheep. In addition, the present study mainly focused on acute responses at 24 h after infection. Whether these molecular alterations persist during chronic infection or eventually recover remains unclear. Future studies incorporating multiple post-infection time points and protein-level validation will further improve our understanding of the temporal progression of *Salmonella*-induced reproductive injury. Furthermore, although transcriptome analysis identified several pathways potentially involved in *Salmonella*-induced orchitis, the specific molecular mechanisms require further functional validation. Because *Salmonella* is an important zoonotic pathogen, the inclusion of both human- and sheep-derived isolates broadens the relevance of this study beyond veterinary medicine. These findings may also provide a basis for future investigations into the reproductive effects of zoonotic *Salmonella* infection across different host species.

## 5. Conclusions

This study identified transcriptomic and molecular changes associated with *Salmonella*-induced male reproductive injury, providing a basis for understanding orchitis, BTB impairment, and spermatogenic dysfunction.

## Data Availability

The raw transcriptome sequencing reads generated in this study have been deposited in the NCBI Sequence Read Archive (SRA) database under BioProject accession number PRJNA1474238.
